# General practitioners’ altered preferences for private practice vs. salaried positions: a consequence of proposed policy regulations?

**DOI:** 10.1186/s12913-015-0777-4

**Published:** 2015-03-25

**Authors:** Jon Helgheim Holte, Birgit Abelsen, Peder Andreas Halvorsen, Jan Abel Olsen

**Affiliations:** Department of Community Medicine,Faculty of Health Sciences, University of Tromsø, 9037 Tromsø, Norway

**Keywords:** Private practice, Salary, Contract, Preferences, General practitioners

## Abstract

**Background:**

General practitioners (GPs) in most high-income countries have a history of being independent private providers with much autonomy. While GPs remain private providers, their autonomous position appears to be challenged by increased policy regulations. This paper examines the extent to which GPs’ preferences for private practice vs. salaried contracts changed in a period where a new health care reform, involving proposed increased regulations of the GPs, was introduced.

**Methods:**

We use data collected from Norwegian GPs through structured online questionnaires in December 2009 and May 2012.

**Results:**

We find that the proportion of GPs who prefer private practice (i.e. the default contract for GPs in Norway) decreases from 52% to 36% in the period from 2009 to 2012. While 67% of the GPs who worked in private practice preferred this type of contract in 2009, the proportion had dropped by 20 percentage points in 2012. Salaried contracts are preferred by GPs who are young, work in a small municipality, have more patients listed than they prefer, work more hours per week than they prefer, have relatively low income or few patients listed.

**Conclusion:**

We find that GPs’ preferences for private practice vs. salaried positions have changed substantially in the last few years, with a significant shift towards salaried contracts. With the proportions of GPs remaining fairly similar across private practice and salaried positions, there is an increasing discrepancy between GPs’ current contract and their preferred one.

**Electronic supplementary material:**

The online version of this article (doi:10.1186/s12913-015-0777-4) contains supplementary material, which is available to authorized users.

## Background

General practitioners (GPs) in most high-income countries have a history of being independent private providers with much autonomy. Rather than direct legal regulations, policy makers have sought to steer GPs’ behavior through financial incentives, including various blends of capitation, fee for service (FFS), bonuses, and - more recently in some countries - pay for performance (P4P) [1,2].

While GPs remain private providers, their autonomous position appears to be threatened by increased regulations [3-5]. In the words of J Fraser [5]: *Internationally, rising costs and increasing consumer and government expectations to monitor quality, contain costs, and integrate health services have increased regulation and reduced the autonomy of GPs and other health care professionals.* Autonomy is known to be a key reason why GPs traditionally have preferred private practice. Consequently, more policy regulations are likely to be perceived as restrictions or impediments to their freedom as self-employed medical providers.

Whether the current remuneration and organization schemes for GPs are in accordance with their preferences is not well documented in the international research literature. In a study of last year medical students and interns in Norway, B Abelsen and J Olsen [6] found that only 20% of young doctors would prefer a fully activity based remuneration contract like the existing one (2/3 FFS and 1/3 capitation). Previous international studies suggest that salaried contracts may be important for recruitment to rural general practice [7-10]. Furthermore, numerous studies show that there is preference heterogeneity across subgroups of doctors, with females in particular having stronger tendency to prefer salaried contracts [6,11,12]. Still, the extent to which GPs’ preferences correspond with the contracts that are currently being offered in general practice remains unclear.

Recruitment and retention of GPs constitute an important health policy concern in many high-income countries where a generational shift takes place simultaneously with an increased demand for GP-services due to an ageing population [6,13]. Clearly, contract type is only one of many factors that influence recruitment and retention [14,15]. Yet, considering that GPs and hospital doctors currently are remunerated and organized in very different ways (i.e. most GPs are private providers, while most hospital doctors work on salaried contracts), it is particularly important to ensure that the “alternative” contracts offered in general practice are in accordance with doctors’ preferences, otherwise it may be difficult to recruit and retain skilled and motivated GPs.

The aim of this paper is to identify the extent to which GPs’ preferences for private practice vs. salaried positions changed in a period where a new health care reform, involving proposed increased regulations of the GPs, was introduced. The reform suggests a new role for the GP including more “out of office” and interdisciplinary work. The proposed regulations, which were introduced in relation to the reform, involved increased obligations in terms of accessibility, meeting attendance, reporting and other administrative tasks. This will provide the municipalities with sufficient legal force to steer GPs out of their office and into more interdisciplinary work, in line with the aim of the reform. Not surprisingly, the proposed regulations were met with heated opposition among GPs and their union. About 3000 GPs (i.e. 75% of all GPs) signed a letter of protest to the Minster of Health and Care Services (http://tidsskriftet.no/article/2505840). Beyond exploring the extent to which GPs’ preferences have changed, we seek to identify factors associated with preferences of contract and to explain GPs’ preferences using data from open-ended questions.

### Institutional context

The Norwegian health care system is organised within two sectors. Municipalities are responsible for primary care, while specialist care is the responsibility of the state (administered by four Regional Health Authorities). The two sectors have different funding mechanisms, as well as different administrative, political and professional cultures [16]. Recently, policy efforts have been made to improve coordination between health care providers [17].

The payment systems for doctors are quite similar to those of other OECD countries [18]. The vast majority of Norwegian general practitioners (GPs) have private practices and receive approximately one-third of their incomes based on capitation (approximately NOK 400 (€50) as a flat rate per patient), paid by the municipalities, and the remaining two-thirds based on FFS. The FFS scheme is a mix of a fixed fee per consultation paid by patients and variable fees paid by the government depending on: the duration of the consultation, whether certain types of examinations and laboratory tests are initiated, and whether the doctor is a specialist in general medicine [19].

Although private practice is the default option for GPs in Norway, other options exists, especially in rural and remote communities. One option is hired practice, in which the municipalities provide offices space, equipment and personnel for a negotiated fee, whereas the GPs still receive capitation and FFS. Other options are salaried positions with or without bonus. Fees for hired practice, salary per hour and design of the bonus component are all negotiated at the municipal level, and to our knowledge there is no central data source, which makes data on these contracts hard to obtain (there are 428 municipalities in Norway). Typically each municipality offers one type of contract, but some municipalities offer salaried positions to young physicians with the option to convert to private practice later. Unlike many other OECD countries [18], the average payment levels are higher among Norwegian GPs than among hospital doctors [6].

## Methods

### Study design and questionnaire

We use data collected through structured online questionnaires in December 2009 and May 2012. All Norwegian GPs were invited to participate in the survey of 2012, while 3270 (81%) were invited to participate in 2009. The remaining 19 percent in 2009 were excluded because they participated in another study at the time. The total number of GPs increased from 4049 to 4305 in the period between the surveys. In the 2009 survey, GPs received e-mailed invitations including an electronic link to the questionnaire. In 2012, they were invited to participate through a postal letter, in which they were asked to access a webpage to answer the online questionnaire. The surveys were planned and executed independently of each other, but some of the questions from 2009 were included in 2012 to enable identification of changes in GPs’ preferences for contract. Results from the 2009 survey have been published previously; see [20].

To map GPs’ current contracts, respondents were asked which of the following options that best describes their practice: (1) private practice in which the GP holds office space, equipment, and employs the staff, (2) private practice in which the GPs hire office space, equipment, and/or staff from the municipality, (3) salary with bonus arrangements, or (4) salary without bonus arrangement. Subsequently, to identify GPs’ preferences for contract, they were asked which of these options they would prefer if they could choose freely. For these questions we used exactly the same wording in 2009 and 2012. The listed alternatives (1) – (4) correspond to the contracts that are in use in Norway. The first option corresponds to the default contract in Norway, while the supplementary options are mainly offered to GPs in rural areas. In this paper, alternative (1) will be referred to as private practice, while alternative (2) will be referred to as hired practice. GPs in private and hired practice are remunerated in the same way, i.e. 1/3 capitation and 2/3 FFS.

In the 2012-survey some additional questions were included to explain GPs’ preference for contract. As a follow-up question on the question regarding preferred contract, respondents were asked: *Would your preferred type of contract still apply if you could be assured that the ability to control working hours, professional development, professional autonomy, and income would be the same in private practice (*or *salary,* when that was the stated preferred contract*)?* We deliberately made this question contingent on ability to control working hours, professional development etc. because these job attributes are known to be important for GPs [14,21].^a^ Thus, the main purpose of this question was to investigate if differences in any other attributes may contribute to explain GPs’ preferences for contract. The respondents were provided with three answering options: ‘*yes*’, ‘*no*’ and ‘*indifferent*’. Those who answered *yes* were asked the following open-ended question: *Why would you still prefer private practice (*or *salary* when that was the stated preferred contract*)?*

Background variables were collected on age, gender, list size, location and specialty attainment in both surveys, as well as for income, working hours, preferred list size and preferred working hours in 2012.

### Data analysis

Descriptive statistics were used to describe the variables of the study, including our main outcomes of interest, i.e. GPs preferences for remuneration in 2009 and 2012. A multinomial logistic regression model was applied to identify factors associated with preferences for contract type, using data from 2012. The dependent variable ‘*preferences for contract*’ was set as categorical with three categories: private practice (1), hired practice (2) and salaried position (3) or (4). The first category (private practice) was treated as the reference in the analysis. The following covariates were included in the multivariate regression analysis: *age*, *gender*, *income*, *specialty attainment*, *location*, *list size*, *working hours*, *discrepancy between preferred and actual list size, discrepancy between preferred and actual working hours*. Data were analyzed on STATA version 12.

Answers to the open-ended question ‘*Why would you still prefer private practice (salaried contract)?*’ were sorted into categories. We used human coding as one of the researchers read every response, identified different themes, and then multi-coded the responses into different categories. Another researcher followed the same procedure and the two agreed on the choice of categories. A representational approach was used in the coding process attempting to map the meaning intended by its source [22].

We use four categories to cover the meanings intended by the responses among those *preferring private practice* (autonomy, more income, aversion to public authorities, other reasons), and five categories to cover meanings intended by the responses among those *preferring salaried positions* (better social security, less administrative work, less employer responsibility, less economic focus, and more stability).

### Ethics

All respondents were informed, through the invitation letter, about the objectives and methods of the study. It was emphasized that participation was voluntary, and that respondents were free to withdraw from the study at any time. The research project was reported to the Data Protection Official for Research (NSD), and was carried out in accordance with existing licenses (e.g. the Personal Data Act). The study does not require approval by the Regional Committees for Medical and Health Research Ethics (REC) (cf. Norwegian Health Research Act).

## Results

### Characteristics

Responses were obtained from 1304 GPs (40% of those e-mailed) in the 2009-survey and 1275 GPs (30% of all Norwegian GPs) in the 2012-survey. In both surveys the respondents were largely representative of Norwegian GPs with respect to age, gender, location and list size, while specialists in general medicine appears to be overrepresented (see Table 1). They worked on average 45 hours per week, but would prefer only 38 hours according to data from 2012. While they preferred to work shorter hours, only 18% of them preferred shorter list size. On average the preferred list size was only 38 patients less than the mean actual list size (1108 vs. 1152). Furthermore, our estimates suggest that GPs earn an average of around NOK 1 050 000 a year, and that there is substantial income variations. (This is gross taxable income after deducting operating costs and national insurance costs (pension, sick pay etc.)).

**Table 1:** **Characteristics**

**Variable**	**Respondents**		**All Norwegian GPs**
	**2009**	**2012**	
	**n = 1 308**	**n = 1 275**	
**Age**	47	48	49^2^
**Female**	36%	37%	35%^2^
**Municipality**			
*<5 000*	13%	16%	14%^1^
*5 000–49 999*	55%	53%	52%^1^
*50 000+*	32%	31%	34%^1^
**Specialty attainment**	66%	70%	55%^3^
**List size** (mean)	1 209	1152	1 182^2^
**Preferred list size** (mean)		1108	
**Discrepancy between preferred and actual list size**			
Fewer patients listed than preferred		18%	
No discrepancy		42%	
More patients listed than preferred		41%	
**Working hours** (weekly mean)		45	
**Preferred working hours** (weekly mean)		38	
**Discrepancy between preferred and actual working hours**			
Work fewer hours than preferred		2%	
No discrepancy		20%	
Work more hours than preferred		78%	
**Current practice**			
*Private practice*	75%	71%	
*Hired practice*	18%	20%	
*Salary with bonus*	4%	4%	
*Salary without bonus*	3%	6%	
**Income** ^**a**^			
< NOK 700 000	-	10%	-
NOK 700 000 – 849 000	-	17%	-
NOK 850 000 – 999 000	-	20%	-
NOK 1 000 000 – 1 149 000	-	17%	-
NOK 1 150 000 – 1 299 000	-	18%	-
NOK 1 300 000 – 1 500 000	-	10%	-
> NOK 1 500 000	-	8%	-
Mean income (SD)^b^	1 051 000 (298 000)		

^1^Statistics Norway (www.ssb.no accessed 24th of March 2011).

^2^ https://helsedirektoratet.no/statistikk-og-analyse/fastlegestatistikk.

^3^ http://legeforeningen.no/Emner/Andre-emner/Legestatistikk/.

Note: Right and left column is copied from [20] ^a^gross taxable income after deducting operating costs and national insurance costs (pension, sick pay etc.) ^b^Mean income is constructed from the mid-points of the selected income range for each GP.

There was no significant change from 2009 to 2012 in the distributions of GPs into different contracts. In 2012, 71% worked in a private practice, 20% worked in a ‘hired practice’, 4% worked in salaried practice with bonuses, while 6% worked in salaried practice without bonuses. Private practitioners (including those who work in hired practice) earn around 15% (NOK 130 000) more than fixed salaried GPs on average (see Additional file 1). However, the variation in income within each contract (e.g. SD NOK 302 000 for GPs in private practice) appears to be much larger than the variation across contracts.

### Preferences for contract

Table 2 shows GPs’ preferences for contract, both the total figures and split within their current contract type. Note the decrease from 52% to 36% in the proportion of GPs who prefer private practice (i.e. the default contract for GPs). The proportion who prefer salary with bonuses increases from 16% to 24%, while the proportion preferring salary without bonuses doubles from 6% to 12%, i.e. the proportion preferring a salaried position increases from 22% to 36%. All these differences are statistically significant.

**Table 2:** **Preferred practice organization and remuneration among Norwegian GPs**

	**Year**	**Preferred practice (row %)**				**Total**	**n**
		**Private practice**	**Hired practice** ^**1**^	**Salary with bonus**	**Salary without bonus**		
		**(95% CI)**	**(95% CI)**	**(95% CI)**	**(95% CI)**		
**Total**	2009	52 (49–55)	26 (24–29)	16 (14–18)	6 (5–7)	100	1294
	2012	36 (33–38)	29 (27–32)	24 (22–26)	12 (10–14)	100	1262
**Current practice**							
Private practice	2009	67	17	13	3	100	964
	2012	47	25	18	10	100	892
Hired practice^1^	2009	10	69	17	4	100	238
	2012	10	57	25	8	100	245
Salary with bonus	2009	2	6	73	19	100	48
	2012	4	4	78	14	100	49
Salary without bonus	2009	2	9	32	57	100	44
	2012	4	8	45	43	100	76

^1^The GP runs a private practice in which premises, equipment, and/or staff are hired from the municipality.

A closer look at preference shifts depending on current contract suggests a particularly strong altering of preferences *within* the private practice group. While 67% of them preferred this contract type in 2009, the proportion had dropped by 20 percentage points in 2012. Among those with hired practice, the support for their current contract dropped from 69% to 57%, with a corresponding preference shift towards salaried alternatives. The highest satisfaction with status quo, as well as the highest preference stability, can be seen in the subgroup *Salary with bonus*. In the subgroup *Salary without bonus* we note an increased preference for having bonus option.

Table 3 shows results from the multinomial logistic regression model estimated on the basis of the sample from 2012, to identify factors associated with preferences for contract. GPs who work in a small municipality and those who have few patients listed are more likely to prefer hired practice over private practice, compared to those with opposing characteristics. Furthermore, GPs who are young; work in a small municipality; have relatively low income; few patients listed, or excessive workload (i.e. more patients listed or more working hours than desired), are significantly more likely to prefer salary over private practice. We find no statistically significant association between gender, working hours or specialty attainment, and preferences for contract in the multivariate regression model.

**Table 3:** **Multinomial regression analysis: factors associated with GPs** ***preferences for practice form***

**Variables**	**GPs preferring** ***hired practice*** **, row %**	**RRR** ^**1**^	**GPs preferring** ***salaried position*** **, row %**	**RRR** ^**1**^
**Age**				
24-37	59% (72/123)	1,564	70% (120/171)	2,315***
38-47	47% (81/173)	1,333	60% (140/232)	2,169***
48-57	43% (102/240)	1,170	36% (78/216)	0,822
>58	38% (78/203)	1	39% (80/205)	1
**Gender**				
Female	48% (114/233)	0,949	62% (198/317)	1,188
Male	43% (219/506)	1	43% (220/507)	1
**Inhabitants in practice municipality**				
0-5 000	80% (57/71)	4,221***	75% (170/226)	6,115***
5 000-14 999	58% (96/165)	1,674**	58% (104/180)	1,300
15 000-49 999	33% (78/238)	0,694*	45% (99/221)	0,658**
>50 0000	38% (102/265)	1	23% (45/197	1
**Specialist**				
No	57% (105/183)	1,300	68% (165/243)	1,163
Yes	41% (228/556)	1	44% (253/581)	1
**Income (NOK)**				
<850 000	57% (86/152)	1,256	72% (168/234)	3,073***
850 000–1 149 999	43% (120/277)	0,929	51% (163/320)	1,593**
>1 149 999	41% (127/310)	1	32% (87/270)	1
**List size**				
0-999	66% (110/166)	2,834***	88% (99/113)	4,357***
1 000-1 199	49% (74/150)	1,735**	59% (101/170)	2,407***
1 200-1 399	41% (84/206)	1,379	38 (99/259)	1,724**
>1 400	30% (65/217)	1	42% (119/282)	1
**Discrepancy between preferred and actual list size**				
Fewer patients listed than pref.	54% (68/125)	0,965	58% (79/136)	0,773
No discrepancy	42% (133/314)	1	47% (158/339)	1
More patients listed than pref.	44% (132/300)	1,348*	52% (181/349)	1,920***
**Working hours**				
<37.6	54% (56/103)	1,371	61% (75/122)	1,153
37.6 – 45	45% (132/293)	1,186	53% (182/343)	1,223
46-52	41% (93/227)	1,002	43% (103/237)	0,952
>52	45% (52/116)	1	48% (58/122)	1
**Discrepancy between preferred and actual working hours**				
Work fewer hours than preferred	67% (8/12)	1,979	79% (15/19)	3,284*
No discrepancy	42% (66/156)		44% (72/162)	1
Work more hours than preferred	45% (259/571)	1,507*	51% (331/643)	1,862***
**Constant**		0,204***		0,074***
Log-Likelihood	−1 114,72			
Number of observations	1 157			

Base level: private practice ***p < 0.01, **p < 0.05, *p < 0.1 ^1^Relative Risk Ratio.

Table 4 shows responses to the follow-up question in the 2012 survey: *Would your preferred type of contract still apply if you could be assured that the ability to control working hours, professional development, professional autonomy, and income, would be the same in private practice (or salaried position)?* The majority of those who initially preferred private practice answered that they would either prefer salary (35%) or be indifferent between salary and private practice (27%) if the listed job attributes would be the same in a salaried position. In contrast, only a small minority of those who initially preferred salary answered that they would change perception if they could be assured that the listed job attributes would be the same in private practice.

**Table 4:** **Would your preferred organization and remuneration system still apply if you could be assured the ability to control working hours, professional development, professional autonomy, and income would be the same in private practice/a salaried position?**

	**Preferred practice (column %)**			
	**Private practice**	**Hired practice**	**Salary with bonus**	**Salary without bonus**
Yes	38	21	78	86
No^1^	35	54	7	8
Indifferent	27	25	14	5
Total	100	100	100	100
N	447	362	300	147

^1^Would prefer salary if preferred system is private or hired practice and private practice if preferred is salary.

Social security/benefits, administrative work and employer responsibility were frequently mentioned reasons why GPs would still prefer a salaried position even if the listed job attributes were equal in private practice (see Table 5). The most frequently mentioned reason for preferring private practice was autonomy, i.e. many respondents did not seem to be assured that autonomy could possibly be the same in a salaried position.

**Table 5:** **Why would you still prefer salary/private practice?** ^**1**^

**Variables**	**n**	**Percent**
**Reasons for preferring Private practice** ^**2**^		
Autonomy	114	46%
More income	34	14%
Aversion to public authorities, bureaucracy etc.	46	19%
Other reasons	18	7%
**Reasons for preferring salaried positions** ^**3**^		
Better social security/benefits	182	50%
Less administrative work	128	35%
Less employer responsibility	81	22%
Less economic focus	43	12%
More stability	49	14%

^1^The respondents were allowed to provide multiple reasons to this question ^2^76 GPs, of those who preferred private practice (N = 248), did not provide an answer to this question. ^3^90 GPs, of those who preferred salaried position (N = 362), did not provide an answer to this question.

## Discussion

GPs’ preferences for private practice vs. salaried positions have changed substantially in the last few years, with a significant shift towards salaried positions. With the proportions of GPs remaining fairly similar across the four contract types, there is an increasing discrepancy between GPs’ current contract and their preferred one.

### Variation in GPs’ preferences: who wants salaried positions?

Young and female GPs are overrepresented among those who prefer salaried positions (see crude percentages in Table 2). These findings are particularly noteworthy considering that a generational shift in the GP population is underway and the share of female doctors is rapidly increasing. The association between gender and preferences for contract is not found to be statistically significant after controlling for other characteristics in the multivariate regression analysis. Thus, gender differences in the other variables (e.g. list size and income) seem to explain why females are more inclined than males to prefer salary. Similar results were obtained in the regression model based on the sample from 2009 [20].^b^

Those who live in small municipalities are considerably more likely to prefer salaried positions. This comes as no surprise since the population base in many small municipalities is too small to make the list patient system with fully activity based remuneration lucrative. Moreover, GPs in small municipalities tend to have more out of office responsibilities, which may be less compatible with the default remuneration system. For this reason, salaried contracts are offered to GPs in rural areas. This finding concurs with results from international studies suggesting that salaried contracts may be particularly important for recruitment to rural general practice [7-10].

As expected, income is an important determinant for GPs contract preferences. For example, those who earn less than NOK 850 000 are three times more likely to prefer salary compared to those who earn more than NOK 1 150 000, (after controlling for differences in hours and all the other independent variables). They might think that the efforts involved in running a private practice do not yield sufficient financial returns.

Furthermore, GPs who have more patients listed than they prefer and work more hours per week than they prefer are more likely to prefer salary over private practice. These findings suggest that the workload they associate with salaried contracts better correspond with their preferred work-life balance, than the workload resulting from an activity based payment system (i.e. 78% work more hours than they prefer and 41% have more patients listed).^c^ Notably, the association between (actual) working hours and preferences for contract is not found to be significant. Thus, the number of hours worked *per se* does not appear to be decisive for GPs contract preferences. The list size, however, remains a significant explanatory factor, i.e. the likelihood of preferring salary decreases with increasing list size.

### Possible explanations for shift in preferences

It seems plausible that the abrupt change in GPs’ preferences for private practice vs salaried positions is influenced by the proposed policy regulations, which were introduced to achieve the aims of a new health care reform, i.e. get the GPs out of their office and into more interdisciplinary work. The proposal challenged the GPs autonomous position through increased obligations in terms of accessibility, meeting attendance, reporting and other administrative tasks. Autonomy is known to be a key reason why GPs traditionally have preferred private practice. Thus, the proposal suggests reducing one of the main attractions associated with private practice. This may have altered the net benefit of being self-employed (rather than salaried) from positive to negative for many doctors. However, other aspect of the reform may also contribute to explain the shift in preferences. Particularly, it might be that the GPs perceive the new responsibilities implied by the reform (i.e. more out of office work and interdisciplinary work), which is more time consuming and difficult to measure, to be less compatible with the current default contract.

The proposal for regulations was released in 2011, but it was not yet adopted at the time of the study. The work of implementing the reform started January 1 2012. However, the content of the reform was already known in December 2009, and the reform was still in an early stage of being implemented in May 2012. Thus, the release and execution of the reform and the proposed regulations cannot be considered as exogenous shocks occurring between the 2009-survey and the 2012-survey. Nevertheless, it seems reasonable to assume that these events may contribute to explain the pronounced shift in GPs’ preferences within a short period of time.^d^

Other factors may also contribute to explain the shift in preferences. First, it appears that the operating costs of running a private practice had increased without being offset by corresponding revenue increases in fees and per-capita grants. Second, younger doctors were in 2011 excluded from the sickness and pension scheme for doctors. While they still have sick pay rights, they now need to sign private insurance for pension. Although these events evoked far less attention and opposition compared to the proposed policy regulations, they were subject of debate in the medical community.

### Discrepancy between preferred and current contract: policy implications

The growing dissatisfaction among GPs with their current default contract might raise policy concern principally in two different ways. First, if it becomes difficult to recruit and retain GPs based on this contract. Second, if it results in suboptimal performance.

So far, recruitment and retention have primarily been a problem in rural municipalities, where alternative payment contracts already exist [23]. The need for GPs is, however, expected to increase substantially in the next years because of a generational shift in the GP population, combined with increased demand for GP-services due to an ageing population [24]. This development will further enhance the challenge of recruitment and retention in the most rural areas. It may also make it difficult to recruit and retain GPs in urban areas, where private practice is offered as the default contract. Anecdotal evidence suggest that GPs in urban areas, particularly young females, recently have quit their jobs because of dissatisfaction with the current organization and remuneration system [25]. This finding is noteworthy since the majority of young doctors are women. However, the growing dissatisfaction with the current default contract is not likely to result in a severe overall shortage of GPs since the number of doctors in Norway exceeds the number of medical positions in hospitals by a wide margin, i.e. many doctors will probably be “forced’ into general practice in the absence of other opportunities.^e^

A more likely scenario, if policy makers refuse to offer contracts in alignment with the diversity in doctors’ preferences, is that general practice, which previously has been a popular specialty in Norway, evolves to loose its recognition and status. This can have a variety of adverse effects. For example, it may become difficult to retain GPs over time, as suggested by LT Kongsvik [25]. Still, winding up a private practice might be costly and take time, so once established it might be for the long run.

Undesirable selection effects can also occur, i.e. it may become difficult to recruit and retain some of those interested and motivated to work as GPs, as they may be deterred by the current contract. This can have negative effects on the productivity and/or the quality of services provided in general practice. More research into this topic is required.

Concerning GPs performance, dissatisfaction with the current contract may potentially have (direct) negative effects, i.e. it might be that those who are dissatisfied with the current contract would have behaved more closely in alignment with policy makers’ objectives if they were allowed to work on salaried contracts. It should be worrisome to policy makers if GPs’ dissatisfaction with their current contract were to create a frustration that might divert them from their professional code of conduct. This aspect seems to be largely ignored in the literature on effects of remuneration systems.

### Expected effects of offering salary

There is a vast literature on how remuneration systems affect physicians’ practice. Most studies find activity based payment schemes (such as FFS and capitation) to be associated with higher output/activity [11,26,27]. It is not fully understood whether the observed association is explained mainly by *selection effects* (i.e. variable remuneration schemes attract workers who are more productive) or *incentive effects* (i.e. variables payment schemes causes workers to be more productive than they otherwise would be under fixed payment schemes). It is hard to separate these effects in field studies which typically lack sufficient data on worker’s preferences and attitudes. However, the existing evidence suggest that both effects play an important role in explaining observed differences in output across variable and fixed remuneration schemes [11,28-30]. Our findings that high income earners and those with many patients listed are more likely to prefer private practice correspond to the findings from previous studies, i.e. activity based remuneration schemes are preferred by workers who are more able and/or more income motivated. Also the findings that those who work more hours and have more patients listed than desired prefer salaried contracts might potentially be explained by differences in productivity, i.e. those who struggle with excessive workload are likely to be less productive (on average) than those who manage to control their workload.

The effect on productivity of offering salary to the GPs who prefer salary depends on the extent to which these doctors are responsive to the financial incentives in the current remuneration scheme, i.e. the magnitude of the incentive effect for this particular group of doctors. Our findings that those who prefer salary over private practice have fewer patients’ listed and lower earnings seem to suggest that this particular group of doctors only to a limited extent is responsive to financial incentives, i.e. it appears that they are willing to trade income for more manageable workload. However, the observed variations in list size and income could also potentially be explained by variations in ability and capacity among GPs.

Neither can we rule out that those who preferred salaried contracts would have become more productive if they had been allowed to work on salaried contracts. To run a private practice against one’s own will is obviously demanding, and it could potentially take a lot of energy and focus away from the core tasks of being a doctor. More research into this topic is required.

Still, reduced productivity might be an inevitable consequence of offering greater variety in contracts, including alternatives with less financial uncertainty, to recruit and retain sufficient number of GPs. The findings that those who work more hours and have more patients listed than they desire are more inclined to prefer salary, suggests that many prefer salary because they want to reduce their current workload. Offering salary with bonus, which seem to be a popular alternative among our respondents, may contribute to reduce the potential risk of negative effects associated with salaried contracts.

Finally, an important caveat should be noted to the literature suggesting that GPs’ productivity is reduced when salaried. These studies are usually based on productivity measures related to consultations and services provided by the GP themselves [26]. It might well be that the seemingly less productive salaried GPs take more time with their patients and reduce unnecessary, i.e. unproductive, referrals to specialists. Hence, when studying how alternative GP payment contracts affect productivity, it is crucial to account for the productivity of the wider health services, i.e. to include the resource use generated by GPs referrals.

### Generalizability

Responses were obtained from 1304 GPs (40%) in the 2009-survey and 1275 GPs (30%) in the 2012-survey.^f^ Compared to similar recent studies these response rates seem satisfactory [31]. Furthermore, and most importantly, our respondents appear to be largely representative with regard to most background characteristics, e.g. age, gender, geographical distribution. Still, we cannot entirely rule out that there are systematic unobserved differences between the 2009-sample and the 2012-sample. For example, it could be an issue that those who were most dissatisfied with the reform, which was about to be implemented in 2012, were most inclined to answering the survey. However, considering that the respondents from the two surveys were almost identical according to all observed characteristics, which are strongly associated with preferences for contract, it does not seem likely that it interferes with our key results to a significant extent.^g^

The recent changes in the organization of GPs in Norway, which appear to have altered their preferences in favor of salaried positions, are similar to changes introduced in a number of other countries where the majority of GPs are private providers [3-5]. Thus, we believe the results from this study are relevant to other contexts and countries. Regardless of what is causing the shift in preferences, our study illustrates the importance of surveying GPs’ preferences for contract type. Notably, we find no other international studies that have examined the extent to which GPs’ preferences for contract type match the existing remuneration and organization systems. More research on this topic is warranted.

## Conclusion

We find that GPs’ preferences for private practice vs. salaried positions have changed substantially in the last few years, with a significant shift towards salary contracts. The emerging pattern of altered preferences is one involving less financial uncertainty. Less than half of the GPs who worked in a private practice (i.e. the default contract for GPs in Norway) in 2012 prefer this contract type. These findings suggest that health authorities may do well in offering more diversity in organization and remuneration schemes.

## Endnotes

^a^The relative importance of the listed job attributes were explored in the 2012-survey using a discrete choice experiment. The results from the experiment will be published in a separate paper.

^b^Results from regression analysis using data from 2009 are presented in Halvorsen et al. [20]. The results from 2009 and 2012 are not directly comparable for several reasons: 1) Income, working hours, discrepancy between preferred and actual list size and discrepancy between preferred and actual working hours were not included as covariates in Halvorsen et al. Data on these variables were not collected in the 2009 survey. 2) The dependent variable ‘preferences for contract’ was dichotomized into private and salary in the analysis based on the 2009-sample, while we use three categories in this paper (as explained in the Methods sections). This approach provides more nuanced information about GPs preferences for the default remuneration system (private practice) vs. other available remuneration schemes (hired practice and salaried positions), and it is feasible to use this subdivision in 2012 since we have roughly 1/3 of the respondents belonging to each group.

^c^In fact, those working fewer hours than preferred also prefer salary, although this is only significant at the 10% level. Such remuneration preferences in this admittedly very small subgroup might be explained by a combination of having recently set up a practice, with too few patients to fill the day, and hence provide an acceptable pay.

^d^Given that the shift in GPs’ preferences indeed was a consequence of the proposed regulations it might be that their preferences have changed after the 2012-survey, because the proposal was modified, to some extent, before it was adopted. Some of the most invasive measures were removed or modified to accommodate the massive opposition from the GPs and their union, but also the adopted regulations (which have been in effect from 1 January 2013) involved increased obligations for the GPs in terms of accessibility, meeting attendance, reporting and other administrative tasks, which are likely to be perceived as impediments to their freedom as self-employed medical providers.

^e^In the proposal for the coordination reform it is stated that the government will ensure that the expected growth in medical positions (i.e. annual net inflow of about 600 doctors to the health service in the next few years) mainly occurs in municipalities, by the means of strict regulation of new medical positions in hospitals.

^f^Approximately 30% of all Norwegian GPs answered each survey (32% in 2009 and 30% in 2012) despite considerable differences in the response rates. The reason is that all Norwegian GPs were invited to participate in the 2012-survey, while only 81% were invited in 2009.

^g^Another possible source of bias is that the 2009-survey was issued through the Norwegian Medical Association’s (NMA) own research institute, *i.e. the Institute for Studies of the Medical Profession*, while the 2012-survey was conducted as an independent survey. Hence, it is conceivable that those who responded to the survey in 2009 are greater supporters of the medical association than those who responded in 2012, meaning that they might have preferences more closely in line with the NMA’s official views. This is a particular concern in the current study since the NMA is known to support the private practice system. However, the vast majority of the Norwegian doctors are members of the NMA (i.e. 97% of all doctors in the age bellow 67 years). Thus, although the degree of support may vary, most GPs support the association in the form of membership.

## Additional file

Additional file 1:
**Earnings according to contract.**

## Abbreviations

CI: Confidence interval
FFS: Fee for service
GP(s): General practitioner(s)
NOK: Norwegian kroner
P4P: Pay for performance
RRR: Relative risk ratio
