# Supplementary material for: General practitioners’ altered preferences for private practice vs. salaried positions: a consequence of proposed policy regulations?
Source: BMC Health Serv Res. 2015 Mar 25;15:119. doi: 10.1186/s12913-015-0777-4 (PMC4417298; doi:10.1186/s12913-015-0777-4)
Supplement: Additional file 1: — Earnings according to contract. [file 12913_2015_777_MOESM1_ESM.doc]

**Additional file 1** Earnings according to contract

| Contract (N) | Mean * | SD |
| --- | --- | --- |
| Private practice (881) | 1 050 243 | 301688 |
| Hired practice (244) | 1 059 893 | 297273 |
| Salaried with bonus (49) | 977 684 | 277451 |
| Fixed salary (75) | 918 293 | 256813 |

*Respondents were provided with six answering categories (i.e. income ranges) in the question concerning income. Mean income is constructed from the mid-points of the selected income range for each GP.
